# O-GlcNAc transferase OGT-1 and the ubiquitin ligase EEL-1 modulate seizure susceptibility in *C*. *elegans*

**DOI:** 10.1371/journal.pone.0260072

**Published:** 2021-11-19

**Authors:** Nirthieca Suthakaran, Jonathan Wiggins, Andrew Giles, Karla J. Opperman, Brock Grill, Ken Dawson-Scully

**Affiliations:** 1 Department of Biological Sciences, Florida Atlantic University, Boca Raton, Florida, United States of America; 2 Department of Neuroscience, The Scripps Research Institute, Jupiter, Florida, United States of America; 3 Center for Integrative Brain Research, Seattle Children’s Research Institute, Seattle, Washington, United States of America; 4 Department of Pediatrics, University of Washington School of Medicine, Seattle, Washington, United States of America; 5 Department of Pharmacology, University of Washington School of Medicine, Seattle, Washington, United States of America; University of South Florida College of Arts and Sciences, UNITED STATES

## Abstract

Neurodevelopmental disorders such as epilepsy and autism have been linked to an imbalance of excitation and inhibition (E/I) in the central nervous system. The simplicity and tractability of *C*. *elegans* allows our electroconvulsive seizure (ES) assay to be used as a behavioral readout of the locomotor circuit and neuronal function. *C*. *elegans* possess conserved nervous system features such as gamma-aminobutyric acid (GABA) and GABA receptors in inhibitory neurotransmission, and acetylcholine (Ach) and acetylcholine receptors in excitatory neurotransmission. Our previously published data has shown that decreasing inhibition in the motor circuit, via GABAergic manipulation, will extend the time of locomotor recovery following electroshock. Similarly, mutations in a HECT E3 ubiquitin ligase called EEL-1 leads to impaired GABAergic transmission, E/I imbalance and altered sensitivity to electroshock. Mutations in the human ortholog of EEL-1, called HUWE1, are associated with both syndromic and non-syndromic intellectual disability. Both EEL-1 and its previously established binding protein, OGT-1, are expressed in GABAergic motor neurons, localize to GABAergic presynaptic terminals, and function in parallel to regulate GABA neuron function. In this study, we tested behavioral responses to electroshock in wildtype, *ogt-1*, *eel-1* and *ogt-1; eel-1* double mutants. Both *ogt-1* and *eel-1* null mutants have decreased inhibitory GABAergic neuron function and increased electroshock sensitivity. Consistent with EEL-1 and OGT-1 functioning in parallel pathways, *ogt-1; eel-1* double mutants showed enhanced electroshock susceptibility. Expression of OGT-1 in the *C*. *elegans* nervous system rescued enhanced electroshock defects in *ogt-1; eel-1* double mutants. Application of a GABA agonist, Baclofen, decreased electroshock susceptibility in all animals. Our *C*. *elegans* electroconvulsive seizure assay was the first to model a human X-linked Intellectual Disability (XLID) associated with epilepsy and suggests a potential novel role for the OGT-1/EEL-1 complex in seizure susceptibility.

## Introduction

GABA neurons serve as a critical component of nervous systems throughout the animal kingdom, ranging from mammals to invertebrates such as *Caenorhabditis elegans* [1–3]. Worms have homologs for approximately 65% of human disease genes in addition to cellular components such as neurons, muscle, gamma-aminobutyric acid (GABA) and acetylcholine (Ach) [4]. The primary inhibitory neurotransmitter in *C*. *elegans* is GABA and the primary excitatory neurotransmitter is Ach. The phase forward movement of the body wall muscles is controlled by these neurotransmitters. Contralateral action of ACh receptors control contraction while contralateral action of GABA receptors control relaxation [5, 6]. In humans, GABA neuronal dysfunction as well as the imbalance between excitatory and inhibitory neurotransmission lead to neurodevelopmental disorders [1, 7–10]. Hence, understanding the mechanism by which GABA neuron function is regulated is critical for understanding nervous system function and neurodevelopmental disorders.

Neurodevelopmental disorders such epilepsy and autism have been linked to an imbalance in the excitation and inhibition (E/I) axis of the central nervous system [11]. This balance can be disturbed through an increase or decrease either in excitatory or inhibitory activity, causing cognitive and behavioral effects. Intellectual disability is associated with a protein called HECT E3 ubiquitin ligase, HUWE1 [12–14]. Mutations in HUWE1 change protein expression levels and are associated with X-linked intellectual disability (XLID) [13]. Experiments conducted in worms and flies indicate EEL-1 and HUWE1 have expanded functions in the nervous system, beyond the scope of early development [14, 15]. There is reported evidence that both an increase and decrease in HUWE1 function can result in intellectual disability, increase hypersensitivity to oxidative stress, and affect genome stability. Importantly, reported cases show epilepsy to be a comorbidity of XLID associated with missense mutations in HUWE1 [14, 16–22]. The *C*. *elegans* homolog of HUWE1 is EEL-1, which was found to function in GABAergic motor neurons as assessed using both pharmacological-behavioral assays and electrophysiological readouts [12]. If HUWE1 is a conserved GABAergic transmission regulator, the increase or decrease in HUWE1 function could affect E/I balance in the brain.

The electroconvulsive seizure assay (ES) is a quantitative tool used to disrupt normal neuronal function and determine how the excitation and inhibition imbalance alters behavior in many systems including *Drosophila*, *C*. *elegans*, and mammals [23–26]. Previous findings determined that EEL-1 is broadly expressed in the nervous system of the worm, including many neurons in the head, mechanosensory neurons, and both GABAergic and cholinergic motor neurons [12]. Although there is no evidence that EEL-1 regulates cholinergic synaptic transmission, there is electrophysiological and behavioral data that indicate EEL-1 affects GABAergic presynaptic transmission [12]. The pharmacological and electrophysiological evidence allowed for the conclusion that reduced inhibitory GABAergic transmission to muscles in *eel-1* mutants lead to an increase in E/I ratio for the *C*. *elegans* motor circuit. The impairment of GABAergic transmission in *eel-1* null mutants likely explain the behavioral abnormalities of reduced locomotion and increased electroshock sensitivity [12]. *unc-25* mutants, which are deficient in GABA synthesis, show similar increased sensitivity to electroconvulsive seizure as *eel-1* mutants [12]. These findings indicate that EEL-1 plays a role in electroshock recovery and in establishing E/I balance.

Following on the discovery that the HECT family ubiquitin ligase EEL-1 preferentially affects GABAergic presynaptic transmission in the *C*. *elegans* motor circuit, recent work has now begun to reveal how EEL-1 regulates GABAergic neuron function [1]. Affinity purification proteomics and biochemistry using *C*. *elegans* identified OGT-1 (O-linked -N-acetylglucosamine (O-GlcNAc) transferase) as the most prominent EEL-1 binding protein. OGT-1 is a conserved glycosyltransferase that modifies protein function in the nucleus, cytosol, and mitochondria [27–29]. OGT is widely expressed in the brain and is localized in presynaptic terminals of mammals [30, 31] and *C*. *elegans* [1]. Although noticeable OGT-mediated O-GlcNAcylation of synaptic proteins has been found, their functional effects in the nervous system is a recent area of active study. OGT regulates mitochondrial motility in neurons and is tied to neurodegenerative diseases [32]. In mice, OGT knockout led to the impairment of AgRP (agouti-related protein) and PVN (paraventricular nucleus) neurons causing an impact on fat metabolism and feeding behavior, respectively [33, 34]. Despite glycosyltransferase activity being the most widely studied OGT activity, recent works suggests that OGT-1 has glycosyl transferase-independent functions in the nervous system [1]. At present, whether OGT-1 affects electroconvulsive seizure is not well explored.

Similar to EEL-1, OGT-1 is expressed broadly in the nervous system, specifically in the cholinergic and GABAergic neurons in the motor circuit and localized in the presynaptic terminals in GABA neurons in *C*. *elegans* [1]. The functional interaction between OGT-1 and EEL-1 that occurs in worms *in vivo* has been biochemically validated, and is a conserved interaction between the orthologous human proteins, HUWE1 and OGT. Automated behavioral assays and pharmacological manipulation of the *C*. *elegans* motor circuit showed that *ogt-1* mutants are hypersensitive to aldicarb similar to *eel-1* mutants. Mutants with impaired cholinergic function accumulate acetylcholine slowly at the synapse upon aldicarb treatment, resulting in slower paralysis and aldicarb resistance compared to wildtype worms. Aldicarb hypersensitivity is rescued by expression of OGT-1 in GABA neurons [1]. *ogt-1; eel-1* double mutants displayed enhanced hypersensitivity to aldicarb [1]. Altogether, these previous findings indicate that OGT-1 and EEL-1 operate in parallel to preferentially affect GABA neuron function. Whether this genetic relationship occurs in other functional contexts remains unknown. Here, we tested the functional interaction between OGT-1 and EEL-1 using our *C*. *elegans* electroconvulsive seizure assay. Our results indicate that OGT-1 and EEL-1 both affect sensitivity to electroshock. *eel-1; ogt-1* double mutants displayed enhanced sensitivity to electroshock. Finally, transgenic pan-neuronal expression of OGT-1 in *ogt-1; eel-1* double mutants rescued enhanced sensitivity to electroshock. These results indicate that OGT-1 and EEL-1 function in parallel pathways to affect electroshock sensitivity.

## Materials and methods

### *C*. *elegans* genetics and transgenics

*Caenorhabditis elegans* used for the initial experiments were standard Bristol N2, loss-of-function *ogt (ok430)*, loss-of-function *eel-1 (bgg1)*, and the double loss-of-function *ogt-1(ok430); eel-1(bgg1)* strains. The *eel-1* protein null, *bggl*, deletes the entire *eel-1* coding sequence, including the HECT ubiquitin ligase domain. The *ogt-1*(*ok430*) mutant is a null allele with a large insertion/deletion that generates a frameshift upstream of the glycosyltransferase domain. For the *ogt-1; eel-1* double mutants, *eel-1* was balanced with *tmC25* [*tmIs1241*] to maintain the strain due to brood size and viability defects. WT animals and single mutants were also balanced as controls. Further rescue experiments were conducted using the *ogt-1(ok430) III; eel-1(bgg1) IV; bggSi2[Prab-3*::*ogt-1a] II* strain. The Bristol N2 strain was ordered from the Caenorhabditis Genetics Center (NIH Office of Research Infrastructure Programs, P40OD010440). All *C*. *elegans* strains were maintained on standard NGM agar plates and seeded with 75μl of OP50 *Escherichia coli*. All initial strains were plated 72 hours prior to picking L4 stage *C*. *elegans* and incubated at 25°C. Approximately 24 hours prior to testing, the L4 worms were then picked and transferred to new NGM agar plates seeded with 75μl of OP50 *E*. *coli* and incubated overnight at 25°C.

### *C*. *elegans* electroconvulsive seizure assay

For the experiments in this study, wt, *eel-1* and *ogt-1* mutants, and *ogt-1; eel-1* double mutants, and OGT-1 pan-neuronal rescue in *ogt-1; eel-1* double mutants were habituated in M9 solution for 30 minutes prior to electric shock. All *Caenorhabditis elegans* strains were run in the electroshock seizure assay as previously outlined [23, 35]. The experimental set-up included Grass SD9 stimulator, Grass SD44 stimulator (utilized as a 3 second timer), dissecting microscope with a camera (Hitachi model KP-D20BU), twelve-inch television monitor, and an HDD and DVR recorder (Magnavox model MDR535H/F7). In 10 mm long clear plastic tubing (Tygon^®^ microbore tubing), 15μl of M9 saline solution was added. Roughly three to six 1-day-old adult worms were picked using a platinum wire pick and placed in those plastic tubes, after which they were incubated for 30 minutes. After incubation, an insulated copper electrode was inserted to both ends of the plastic tube. A 1cm gap was measured in between both electrodes. The electrodes were fastened with alligator clips to a square-pulse generating stimulator (Grass, SD9) that delivered a 3 second, 47V shock. Approximately six replicates per genotype were done.

### Electroconvulsive seizure assay video recovery time recording

Video capture via a dissecting microscope camera (Hitachi model KP-D20BU) was initiated 10 seconds prior to the administration of the shock. Synchronized adults (L4) had a baseline behavior recorded for 10 seconds prior to administration of the electric shock. Speed was normalized to M9 buffer to examine the effects of a given genotype on locomotion. The mean speed was calculated every minute and corresponded to the total amount of sinusoidal wave-like swimming per worm. Video capture continued for up to 5 minutes after the shock had taken place. Due to electrolysis, bubbles formed on either end of the plastic tube. Data points corresponding to the recovery times of each *Caenorhabditis elegans* were then collected. Recovery time was defined as the point at which three normal sinusoidal wave-like swimming movement resumed following the initiation of the shock. Each individual worm was manually scored the minute they resumed sinusoidal motion. *C*. *elegans* were excluded from analysis if they intersected the peripheral bubbles. Those animals that did not recover after the shock were excluded from the time to recovery analysis and included in the percent of animals’ recovery analysis. All recovery times and percent of animals that recovered upon electroshock are shown as averages for each genotype.

### Pharmacological manipulations

Drugs were dissolved directly into M9 saline solution and 15μl of solution was aliquoted into transparent plastic tubing. Animals were incubated in drugs of interest for 30 minutes prior to electric shock. The drug tested was Baclofen and it was obtained from Sigma-Aldrich, St. Louis, MO, USA.

### Statistical analysis

**Fig 1:**
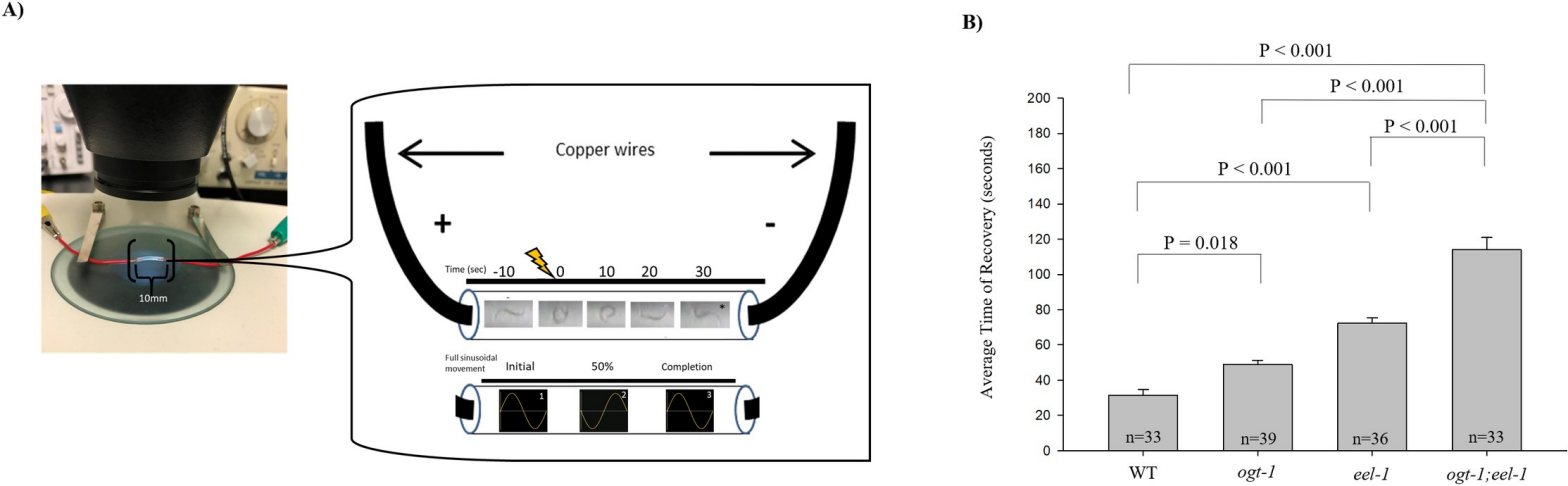
*ogt-1; eel-1* double mutants have heightened sensitivity to electroshock. (A) Representative close-up images of the experimental tube and electroshock assay readout of wild-type (N2) *C*. *elegans*. The tube contains a liquid M9 solution with copper wire on either side of the tube. There are 3–6 worms for each trial of electroshock administration. Shown in the experimental tube are freeze-frame images of a single N2 worm. The images are still frames in 10 second increments from a video of raw data seen in S1 video. These images are taken before the shock in which the animal moves in a sinusoidal wave pattern (denoted in -10 seconds), during the shock where the worm is exhibiting unilateral body bends or paralysis that characterizes the convulsion phase (denoted at 0 seconds), and after the shock where the animal fully recovers locomotion within ~30 seconds as denoted by the asterisk. Sinusoidal movement rate was calculated based on 1 full sinusoidal movement/10 seconds and standardized to 1 minute. From left to right, image 1 shows the starting point of movement, image 2 is when the worm completed 50% of the movement, and image 3 shows a worm that has reached 1 full sinusoidal movement. (B) Shown are each genotype ran through the electroconvulsive seizure assay for 3 seconds at 47V in M9 solution. Average time of recovery was significantly increased in *ogt-1; eel-1* double mutants (n = 33) when compared to wildtype (n = 33), *eel-1* null mutants (n = 36), and *ogt-1* null mutants (n = 39). Error bars represent standard error of the mean. Significance was determined using a One-Way ANOVA with a Bonferroni test–pairwise. Significance between columns were denoted using a *p<0.05, **p<0.01, ***p<0.001. Data was collected from 6/14/19-6/20/19, separate from Fig 2A.

**Fig 2:**
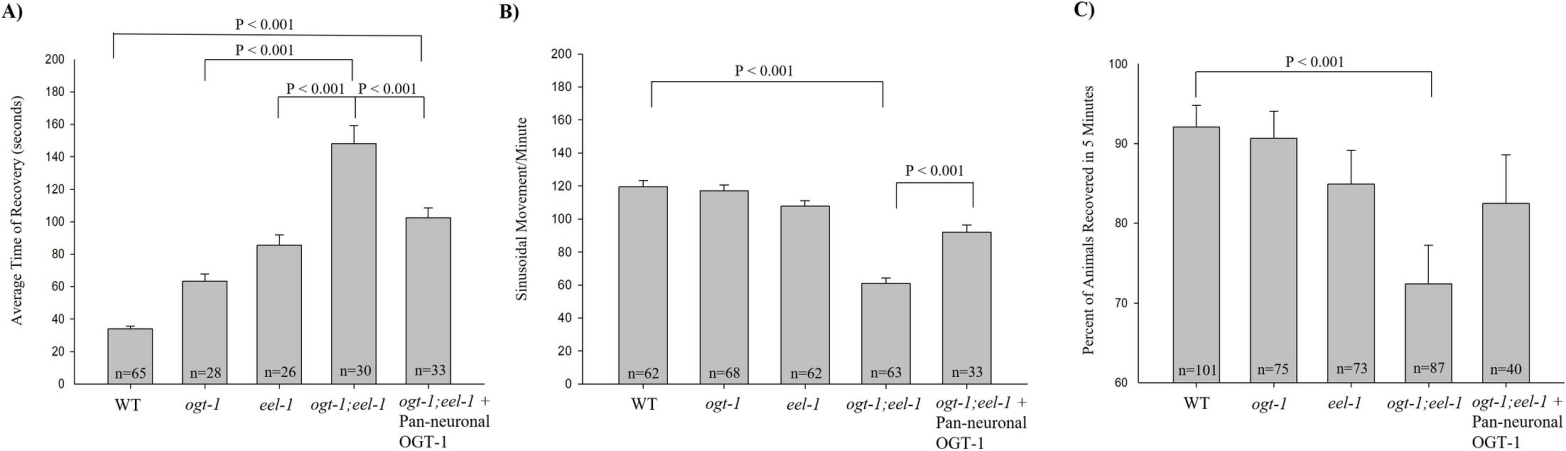
*ogt-1; eel-1 +* Pan-neuronal OGT-1 have decreased sensitivity to electroshock and improved locomotion. Shown are each genotype ran through the electroconvulsive seizure assay for 3 seconds at 47V in saline control group. (A) Average time of recovery was significantly decreased in *ogt-1; eel-1* + Pan-neuronal OGT-1 worms, *Ogt-1(ok430) III; eel-1(bgg1) IV; bggSi2[Prab-3*::*ogt-1a] II* (n = 33), in comparison to *ogt-1;eel-1* double mutants (n = 30) but not significantly different from *eel-1* null mutants (n = 26). Wildtype (n = 65) recovered significantly earlier than the *eel-1* null mutants (n = 26) and *ogt-1* null mutants (n = 28). Error bars represent standard error of the mean. Significance was determined using a One-Way ANOVA with a Student-Newman-Keuls Method- pairwise (A,C) and a Bonferroni test (B)–pairwise.(B) The average sinusoidal pre-shock movement depicted displayed the forward speed of *ogt-1; eel-1* + Pan-neuronal OGT-1 worms, *Ogt-1(ok430) III; eel-1(bgg1) IV; bggSi2[Prab-3*::*ogt-1a] II (n = 33)* to be similar to *eel-1* null mutants (n = 62), but significantly faster than *ogt-1;eel-1* double mutants (n = 63). Wildtype (n = 62), *eel-1* null mutants (n = 62), and *ogt-1* null mutants (n = 68) move at a similar sinusoidal speed. (C) Average percent of animals that recovered in 5 minutes for the *ogt-1; eel-1* null mutants (n = 87) was significantly lower than wildtype (n = 101), while the *ogt-1; eel-1* + Pan-neuronal OGT-1 worms, Ogt-1(ok430) III; eel-1(bgg1) IV; bggSi2[Prab-3::ogt-1a] II (n = 40) did not significantly differ from the percent of wild-type (n = 101) animals that recovered. Significance between columns were denoted using a *p<0.05, **p<0.01, ***p<0.001. Data was collected from 6/14/19-6/20/19, 6/24/19-7/9/19, and 10/14/19.

Average time of recovery and average sinusoidal pre-shock movement data for wildtype, *eel-1* null mutants, *ogt-1* null mutants, *ogt-1; eel-1* double mutants and OGT-1 pan-neuronal rescue animals were analyzed using a One-Way Anova on Ranks with a Bonferroni Test—pairwise (Figs 1B and 2B). Average time of recovery and average percent recovered for wildtype, *eel-1* null mutants, *ogt-1* null mutants, *ogt-1; eel-1* double mutants, and OGT-1 pan-neuronal rescue in *ogt-1; eel-1* double mutant strains were analyzed using a One-Way Anova on Ranks with a Student-Newman-Keuls Method-pairwise (Fig 2A and 2C). All bar graphs represent a mean ± SEM (error bars) and the letters denote statistical groupings. Significance between columns were denoted using a *P<0.05, **P<0.01, ***P<0.001, and ns = not significant. Outliers greater than the 75th percentile value + 1.5*IQR and outliers less than the 25 percentile value—1.5*IQR were removed.

## Results

### *C*. *elegans* behavioral response to electric shock

We have developed a model of seizure in *C*. *elegans* through the use of an electric shock to induce convulsions [23, 35]. This model of seizure is similar to practices in other invertebrate and vertebrate models [26, 36]. Worms were initially placed in a transparent plastic tube with M9 liquid saline solution (Fig 1A). A copper wire was plugged into both ends of the tube (10 mm apart from one each other) and connected to a square-pulse generating stimulator. The voltage of 47V was used as this was previously found to be the maximum voltage at which recovery occurred [35]. Through this method, we are able to assess approximately three to six worms per tube and record animals in real-time with a camera. A brief electric shock is applied for a period of 3 seconds, worms show paralysis and elongation in our “Electroshock Induced Seizures in Adult *C*. *elegans*” Bio-Protocol (S1 Video and Fig 1A). Following immediately afterward are slow unilateral body-bends and convulsions, which are unilateral body bends with muscle twitching. Upon removal of the electric shock, a rapid recovery where most animals resume sinusoidal swimming movement is displayed (Fig 1A). Shown in Fig 1A is an example of a still image of a wild-type worm assessed before (-10 sec), during (0 sec), and after the electric shock (10 sec, 20 sec, 30 sec). Asterisks highlight time frames in which the worm has recovered from electric shock. The still images for the real time video in Fig 1A can be seen in our S1 video [23]. Consistent with several prior studies [23, 35], our electroconvulsive seizure assay indicates that electric shock induces paralysis and convulsions in *C*. *elegans*.

### OGT-1 and EEL-1 function in parallel to regulate electroshock responses

We have previously established that electroshock induced paralysis is a sensitive behavior to E/I balance in the worm motor circuit [23, 35]. Earlier work done in the Dawson-Scully lab showed *unc-25/GAD* mutants that lacked inhibitory GABAergic transmission had longer time to recover, indicating an increased electroshock sensitivity [12]. Prior genetic work done in the Grill Lab used automated, behavioral pharmacology assays to show that OGT-1 and EEL-1 acted in parallel to regulate GABAergic neuron function [1]. Since OGT-1 binds EEL-1, is expressed in GABAergic motor neurons, and localizes to GABAergic presynaptic terminals, we tested how OGT-1 affects motor circuit function through the electroshock assay (Fig 1A). To investigate the biological relationship between OGT-1 and EEL-1 proteins we evaluated *ogt-1* null mutants, *eel-1* null mutants, and *ogt-1; eel-1* double mutants using our electroshock assay. The time to recovery of *ogt-1* (48.95 seconds), *eel-1* (78.72 seconds), and *ogt-1; eel-1* double mutants (113.94 seconds) were significantly longer than wildtype (31.48 seconds) (P<0.05, Fig 1B). *ogt-; eel-1* double mutants also showed enhanced increases in recovery time from electroshock compared to single mutants (Figs 1B and 2A). Previous studies found reduced presynaptic GABAergic transmission in *eel-1* null mutants resulted in increased sensitivity to electroshock [12]. Our results are consistent with these previous findings. Building upon these results, we now show that *ogt-1* mutants have increased sensitivity to electroshock compared to wild-type worms. Moreover, enhanced deficits in recovery from electroshock indicate that EEL-1 and OGT-1 function in parallel to affect electroshock sensitivity.

### OGT-1 pan-neuronal expression in *ogt-1; eel*-1 double mutants rescues sensitivity to electroshock

Our experiments have shown that *ogt-1; eel-1* double mutants have heightened sensitivity and recovery time to electroshock-induced paralysis compared to wild-type worms as well as *ogt-1* and *eel-1* single mutants (Figs 1B and 2A). Our current experiments were based on previous findings that reported *ogt-1* functions in GABA neurons to regulate aldicarb sensitivity and enhances *eel-1*. We ran the *ogt-1* and *eel-1* null mutants through our electroshock assay because this assay was reported to be highly sensitive to mutations that reduce inhibitory GABAergic transmission to muscles. To confirm that loss of OGT-1 affects motor circuit function and electroshock sensitivity, we performed transgenic rescue experiments. To do so, we used a pan-neuronal promoter to expression OGT-1 throughout the nervous system of *ogt-1; eel-1* double mutants. These animals were then subjected to the electroshock assay. Average time of recovery from electroshock was significantly decreased in *ogt-1; eel-1* double mutants expressing pan-neuronal OGT-1 (102.42 seconds) compared to *ogt-1; eel-1* double mutants (148.03 seconds) (Fig 2A). There remained a significant difference in recovery time between *ogt-1; eel-1* double mutants expressing pan-neuronal OGT-1 (102.42 seconds) and wild-type worms (33.85 seconds). This is because these animals still lack EEL-1 function and *eel-1* single mutants have increased ES sensitivity (Fig 2A). A similar behavior was seen through the assessment of locomotion, where an improvement as well as increase in sinusoidal pre-shock movement in the OGT-1 pan-neuronal rescue in *ogt-1; eel-1* double mutants (92 sinusoidal movements/minute), compared to the *ogt-1; eel-1* double mutants (60.95 sinusoidal movements/minute) (P<0.001, Fig 2B). This indicates that expression of OGT-1 in the nervous system rescues enhanced locomotion defects in *ogt-1; eel-1* double mutants. However, there is still a significant decrease in locomotion with the OGT-1 pan-neuronal rescue in *ogt-1; eel-1* double mutants (92 sinusoidal movements/minute) when compared to wild-type worms (107.71 sinusoidal movements/minute) (Fig 2B). These results indicate that OGT-1 interacts with EEL-1 to affect both electroshock sensitivity and locomotion. Both findings are consistent with the notion that OGT-1 and EEL-1 may be required for the motor circuit to obtain E/I balance.

### OGT-1 and EEL-1 function in parallel to regulate locomotion

To further examine how genetic interactions between OGT-1 and EEL-1 affect motor circuit function, we evaluated locomotion in M9 buffer. Previous work analyzed locomotion in M9 buffer by monitoring swimming speed using Multi-Worm Tracker [1]. These studies found that swim speed trended towards impaired locomotion for *eel-1* mutants and mildly defective in *ogt-1* mutants compared to wild-type worms [1]. *ogt-1; eel-1* double mutants showed an enhanced reduction in swim speed compared to *eel-1* single mutants [1]. Here, we evaluated locomotion by manually calculating sinusoidal movement rates prior to administration of the electroshock. We observed locomotion abnormalities in *ogt-1; eel-1* double mutants compared to wildtype, and swim speed trended towards impaired locomotion in *ogt-1* and *eel-1* single mutants. The double mutants showed the lowest pre-shock sinusoidal movement rate of 60.95 sinusoidal movements/minute in comparison to 119.52 sinusoidal movements/minute for wildtype (P<0.001, Fig 2B). This was an enhanced decrease in locomotion compared to *eel-1* or *ogt-1* single mutants. These findings are consistent with OGT-1 and EEL-1 forming a complex, and demonstrate that OGT-1 and EEL-1 act in parallel to affect locomotion.

### OGT-1 and EEL-1 affect total percent of recovered animals following electroshock

We have shown that the average time of recovery from electroshock for the *ogt-1; eel-1* double mutants (113.94 seconds) was significantly longer than wildtype (31.48 seconds) (Fig 1B). To determine whether recovery was also compromised, we calculated the average number of worms that were able to recover following administration of electroshock, and present data as the percentage of animals that recover from electroshock. Around 92.08% of wild-type worms and 72.41% of the *ogt-1; eel-1* double mutants recovered from an electroconvulsive seizure (Fig 2C). Hence, the total percent of animals that recovered upon electroshock was significantly reduced in the *ogt-1; eel-1* double mutants, in comparison to wildtype (P<0.001, Fig 2C). Interestingly, 92.08% of the wildtype and 82.5% of the OGT-1 pan-neuronal rescue animals recovered upon electroshock administration, displaying no significant difference between them (P = 0.448, Fig 2C). In addition, the OGT-1 pan-neuronal rescue animals trended towards being rescued but did not reach significance when compared with the *ogt-1; eel-1* double mutants (P = 0.222, Fig 2C). These results show that OGT-1 and EEL-1 function in parallel to affect the fitness of animals following electroshock.

### OGT-1 and EEL-1 affect electroshock recovery through altered GABAergic neuron function

Our previous findings have shown that genetic manipulation of GABA neuron function alters electroshock responses [1, 35]. Loss-of-function mutants for *unc-25* (which encodes the GABA biosynthetic enzyme GAD) and *unc-49* (which encodes the GABA_A_ receptor) have decreased inhibitory GABAergic transmission to the muscles, and display significantly longer time to recovery following electric shock compared to the wild-type animals [35]. Our previous electrophysiological results showed that *eel-1* mutants have impaired GABAergic motor neuron function [12]. Consistent with partially impaired GABAergic transmission, *eel-1* null mutants displayed impaired locomotion and heightened sensitivity to electroshock [12, 35] (Figs 1B and 2A).

**Fig 3:**
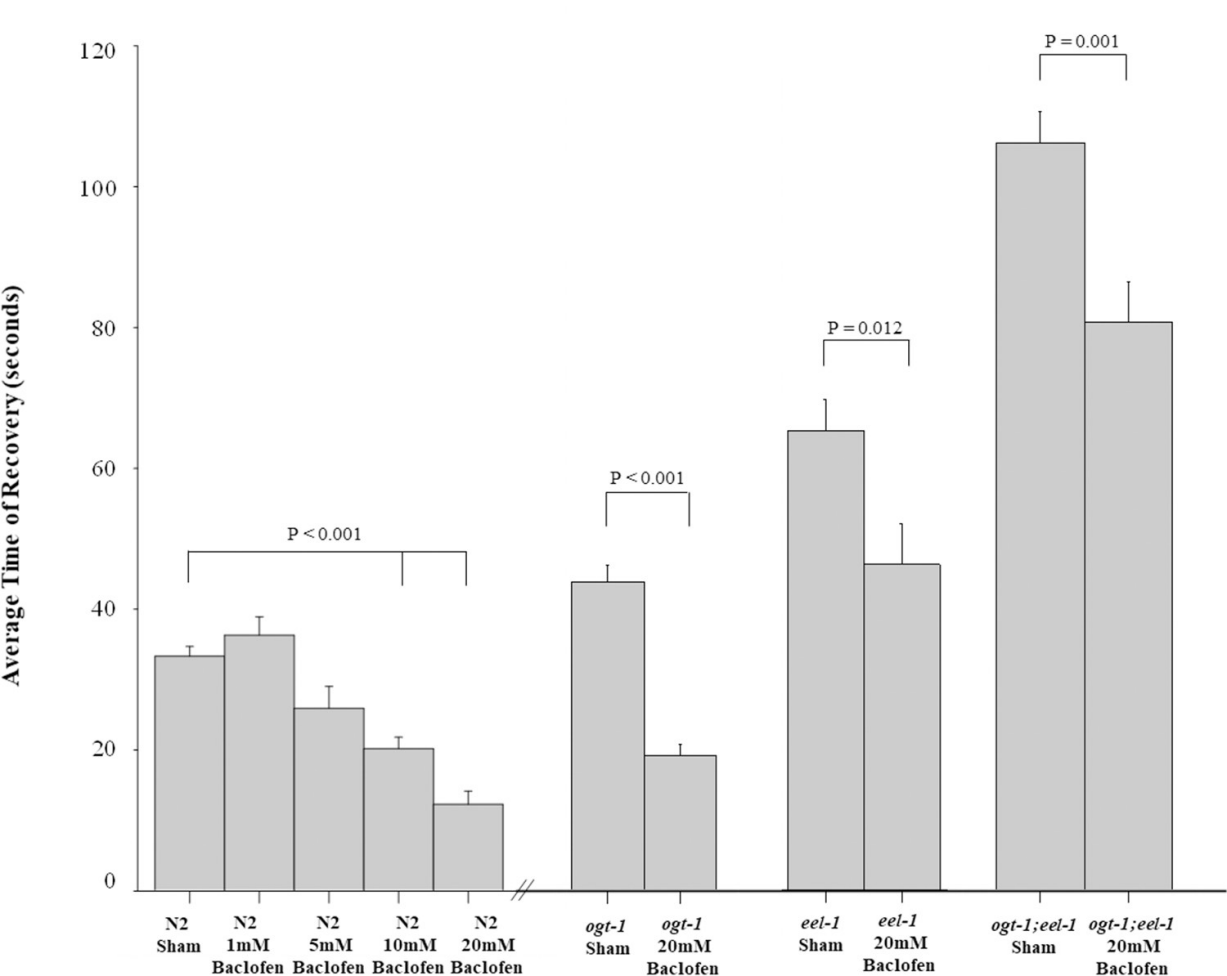
Increased sensitivity to electroshock in *ogt-1; eel-1* double mutants is improved with application of GABA receptor agonist Baclofen. Shown are each genotype ran through the electroconvulsive seizure assay for 3 seconds at 47V in Baclofen. (A) The wildtype animals displayed a trending significant decrease in recovery time upon increasing doses of 5mM (n = 14), 10mM (n = 23), and 20mM Baclofen (n = 14), compared to these animals in the sham, M9 saline control group (n = 57). Upon 20mM of Baclofen administration, average time of recovery was significantly reduced in *ogt-1; eel-1* worms (n = 18), *ogt-1* null mutants (n = 21), *eel-1* null mutants (n = 15) compared to *ogt-1;eel-1* worms (n = 20), *ogt-1* null mutants (n = 25), *eel-1* null mutants (n = 22) in sham, M9 saline. Significance was determined using a One-Way ANOVA with a Student-Newman-Keuls Method- pairwise. The error bars are SEM and significance between columns were also denoted using a *p<0.05, **p<0.01, ***p<0.001.

To pharmacologically assess how altering GABAergic transmission affects recovery from electric shock, we evaluated the effects of Baclofen, a GABA receptor agonist. We tested wildtype animals, *ogt-1* mutants, *eel-1* mutants, and *ogt-1;eel-1* double mutants in sham M9 solution or Baclofen. The dose response curve of Baclofen on wildtype animals showed reduced electroshock responses with increasing doses (Fig 3). Wild-type animals had significantly improved recovery from electroshock at 5mM (P = 0.027, 25.93 seconds), 10mM (P<0.001, 20.22 seconds), and 20mM of Baclofen (P<0.001, 12.29 seconds) compared to those in the sham, M9 solution (34.45 seconds). The maximum effect on wild-type animals occurred at 20mM Baclofen. Higher concentrations of Baclofen could not be used because they affect the animal’s native locomotion and lead to paralysis in the absence of electroshock (data not shown). *ogt-1* single mutants in 20mM Baclofen (18.90 seconds) showed significant reduction in recovery time from electroshock compared to sham treatment (P<0.001, 43.08 seconds, Fig 3). Similarly, *eel-1* null mutants treated with 20mM Baclofen (45.27 seconds) had significantly reduced time to recovery compared to *eel-1* sham animals (P = 0.012, 63.86 seconds, Fig 3). Finally, average time of recovery from electroshock was significantly reduced in *ogt-1; eel-1* double mutants in 20mM of Baclofen (80.39 seconds) compared to these animals in saline (P = 0.001, 105.80 seconds, Fig 3). These pharmacological results suggest that OGT-1 and EEL-1 effects on electroshock sensitivity are a result of impaired GABA neuron function.

## Discussion

In this study, we have utilized and validated the electroconvulsive seizure assay to investigate seizure-like behavior in wild-type *C*. *elegans*, *eel-1* mutants, *ogt-1* mutants, and *ogt-1; eel-1* double mutants (Fig 1A and 1B). The behavioral response after an electroshock has been defined as “seizure like” based on paralysis of wild-type animals that was followed by hypercontraction of muscles and unilateral body bends. This physical seizing is in accordance with its response to electroshock in other seizure models [25, 26, 37]. Previous studies utilized *unc-25* and *unc-49* loss-of-function mutants that lacked inhibitory GABAergic neurotransmission to muscles. These classic hyperexcitable mutants had significantly delayed recovery following electroshock compared to wild-type animals [23, 35, 38–41]. These previous findings demonstrate that GABAergic neurotransmission plays a role in the time to recovery after electroshock. Our current study now further validates the role of GABA neuron function in electroshock responses, and the validity of this rapid, reliable approach in assessing genetic effects on seizure in *C*. *elegans*.

There is much that remains unknown about the molecular mechanisms that preferentially affect GABAergic transmission. In mammals, there is less known about presynaptic GABA-specific regulators but there are proteins such as synapsins that differentially impact inhibitory transmission compared with excitatory transmission [42, 43]. Although *C*. *elegans* also has few proteins known to regulate presynaptic GABA function, they do have core presynaptic proteins with conserved roles in neurotransmission in the motor circuit. The worm motor circuit also serves as a simple model circuit where a balance between excitatory cholinergic and inhibitory GABAergic neuron function is required for appropriate behavioral outcomes [6, 44]. Hence, the worm motor circuit has proven to be of value in identifying key molecules that regulate GABA neuron function. The focus of our study is on the HECT family ubiquitin ligase EEL-1 and its binding protein OGT-1.

Our interest in exploring how both EEL-1 and OGT-1 regulate electroshock responses, electroconvulsive seizure and GABAergic neuron function was increased due to the genetic links between the EEL-1ortholog HUWE1 and XLID in mammals [14]. Known cases note that epilepsy is a comorbidity with ID in patients harboring mutations in *HUWE1* [14, 16–20, 22, 45–47]. Prior to beginning our study, it was known that OGT-1 and EEL-1 interact biochemically and genetically in *C*. *elegans* neurons *in vivo* [1]. In fact, this interaction is conserved between HUWE1 and OGT, the orthologous human proteins. Previous results from genetic analysis utilized an automated behavioral assay and pharmacological manipulation of the *C*. *elegans* motor circuit via an aldicarb assay to show that both EEL-1 and OGT-1 act in parallel to affect locomotion as well as GABA neuron function [1]. Our electroshock assay serves as a complement to these previous approaches, and offers an independent method for altering cellular excitability in worms. This allowed us to quantify the effects of EEL-1 and OGT-1 on both time to recovery from electroshock, percent of recovery following electroshock, and in basal locomotion in liquid.

Prior to electroshock induction, we calculated the sinusoidal movement rates and showed mild movement abnormalities in *eel-1* single mutants and more substantial, significant defects in locomotion in *ogt-1; eel-1* double mutants in comparison to wildtype (Fig 2B). Our findings are consistent with the findings of automated analysis of swimming speed on *ogt-1; eel-1* double mutants [1]. Interestingly, upon pan-neuronal rescue of OGT-1 in the *ogt-1; eel-1* double mutants, the sinusoidal movement rate was improved (Fig 2B). Currently, the function of OGT-1 in *C*. *elegans* and mammals has only been minimally explored. Through previous affinity purification proteomics done in *C*. *elegans*, OGT-1 was identified as a prominent EEL-1 binding protein. Biochemical data indicate that OGT-1 binds EEL-1 in neurons *in vivo*. In fact, like EEL-1, OGT-1 is in the motor circuit of the nervous system and localizes to GABAergic presynaptic terminals. Our results showing that EEL-1 and OGT-1 function in parallel to regulate recovery and viability following electroshock and locomotion is consistent with EEL-1 and OGT-1 functioning in the same protein complex. Other examples of these types of biochemical and genetic relationships include interactions between FSN-1, GLO-4, RAE-1, ANC-1 and PPM-2 which are in protein complexes together and function in parallel to regulate axon and synapse development [48–51]. Thus, there is precedent for parallel genetic interactions with molecules from shared protein complexes.

To evaluate motor circuit function, pharmacological assays based on the acetylcholine esterase inhibitor aldicarb are often employed [1, 52]. Aldicarb application causes hyperexcitability, excess muscle contraction and eventual paralysis in *C*. *elegans*. Previous studies have found that aldicarb hypersensitivity in *ogt-1* mutants is enhanced by loss of *eel-1* function [1]. Since EEL-1 is a known regulator of presynaptic GABAergic transmission, the OGT-1/EEL-1 complex and effects on aldicarb sensitivity have been suggested to occur through effects on GABAergic transmission [1]. Our current findings are the first to show that *ogt-1; eel-1* double mutants have increased sensitivity to electroshock (Fig 2A). This is supported by the significantly lower percentage of *ogt-1; eel-1* double mutants that recover following electroshock (Fig 2C). These enhancer effects on electroshock sensitivity can be rescued by transgenic pan-neuronal expression of OGT-1 (Fig 2A and 2C). In addition, our pharmacological assessment revealed that increasing GABA receptor activity via Baclofen reduced susceptibility to electroshock for *ogt-1* single mutants, *eel-1* single mutants, and *ogt-1;eel-1* double mutants. These findings with Baclofen administration are consistent with OGT-1 and EEL-1 affecting GABA neuron function to impact seizure susceptibility. Our findings here are also consistent with prior studies that showed mutants lacking *unc-25/GAD* and *unc-49/GABA*_*A*_ have altered electroshock responses [35]. Thus, we provide further evidence that our *C*. *elegans* electroshock seizure assay detects deficits in GABA neuron function, and is sensitive to genetic and pharmacological manipulations that alter E/I balance in this simple model circuit. The implications of our findings could be broadly relevant in other systems, since the OGT-1/EEL-1 complex is a conserved complex that forms been orthologous human proteins [1].

Our studies have now quantified and confirmed that OGT-1 and EEL-1 have shared functions in the *C*. *elegans* nervous system via effects on three readouts: 1) electroshock sensitivity, 2) percent of animals’ recovery following electroshock, and 3) locomotion. Our findings encourage exploration of the relationship between OGT and HUWE1 in other functional contexts where these molecules have known roles, such as oncogenesis, neural progenitor proliferation [53–56] and mitochondrial function [32, 57–59]. Our findings are also consistent with prior clinical studies that showed genetic changes in *HUWE1* are associated with neurodevelopmental disorders that have seizure/epilepsy as a comorbidity [14]. Similar to *HUWE1*, genetic studies have identified mutations in *OGT* (7 different mutations from 11 individuals) with ID [60–64]. In fact, familial inheritance shows *OGT* mutations segregate with ID. Consistent with our results showing OGT-1 affects electroconvulsive seizure in our *C*. *elegans* model, epilepsy/seizures were identified in patients with missense mutations in *OGT* [65]. Our studies demonstrate that OGT-1 may have a novel molecular function in seizure susceptibility, as part of the complex it forms with EEL-1. Our pharmacological results suggest that EEL-1 and OGT-1 affect GABA neuron function to influence electroshock-induced seizure. Future studies will be needed to further unravel the molecular and genetic mechanisms by which OGT-1 and EEL-1 influence seizure.

## Supporting information

S1 Video Electroshock assay setup and annotated footage of the wild-type worms. This video shows an example of the current experimental setup, followed by video data of our wildtype worms before and after electroshock administration. There are five worms in the experimental tube containing saline solution, points of recovery determination are displayed for all worms.
(MP4)
